# New aspects of improving the performance of WO_3_ thin films for photoelectrochemical water splitting by tuning the ultrathin depletion region†

**DOI:** 10.1039/c8ra08875f

**Published:** 2019-01-08

**Authors:** Jiajie Cen, Qiyuan Wu, Danhua Yan, Wenrui Zhang, Yue Zhao, Xiao Tong, Mingzhao Liu, Alexander Orlov

**Affiliations:** Department of Materials and Science Engineering, Stony Brook University Stony Brook New York 11794 USA alexander.orlov@stonybrook.edu; Center for Functional Nanomaterials, Brookhaven National Laboratory Upton New York 11973 USA mzliu@bnl.gov; Department of Chemistry, Stony Brook University Stony Brook New York 11794 USA

## Abstract

In this work, we explored a facile, scalable and effective method for substantially enhancing photocurrent and incident-photon-to-current efficiency of WO_3_ thin-film photoanodes by a mild reduction treatment under low oxygen pressure. Experimental data from photoelectrochemical and electrochemical impedance spectroscopies have shown that such treatment can increase the charge carrier density on WO_3_ photoanode surfaces resulting in improvements in hole collection efficiency and reduction in charge recombination. Despite a much thinner layer of WO_3_ (about 500 nm) compared to those in other published studies, the electrodes exhibited an ultra-high photocurrent density of 1.81 mA cm^−2^ at 1.23 V *vs.* RHE. This current density is one of the highest ones among WO_3_-based photoanodes described in literature. The proposed surface modulation approach offers an effective and scalable method to prepare high-performance thin film photoanodes for photoelectrochemical water splitting.

## Abstract 2

Surface modulation approach offers an effective and scalable method for high-performance WO_3_ photoanodes.

## Graphical abstract

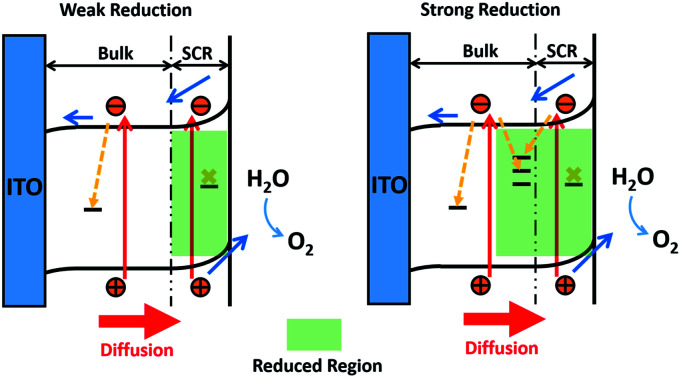

## 1. Introduction

Water splitting in photoelectrochemical (PEC) cells is potentially the most promising method for converting solar energy into chemical energy. Tungsten trioxide (WO_3_), which is an n-type semiconductor, is one of the most promising candidate materials for PEC water oxidation.^1^ This semiconductor has an indirect band gap of 2.5–2.8 eV while being among only a few visible-light-responsive photocatalysts that exhibit outstanding stability in acidic electrolytes (pH ≤ 4). In contrast, other visible-light activated photoanode materials, such as BiVO_4_ and α-Fe_2_O_3_, are less stable under the same acidic conditions. This is important as photoanodes with significant stability in acidic environments (*e.g.* WO_3_) could be utilized in acidic water electrolyzers based on polymer electrolyte membranes, which overcomes many of the disadvantages of the conventional alkaline electrolyzers.^2^ The disadvantages of WO_3_ based anodes stem from their indirect band gap. As a result, WO_3_ exhibits a weak light absorption in visible range (*α* = 10^4^ to 10^5^ cm^−1^), which is about one order of magnitude lower than that of other visible light photocatalysts, such as Fe_2_O_3_ and BiVO_4_. This disadvantage can be potentially offset by having a relatively long (∼500 nm) hole diffusion length in the WO_3_ materials, thereby conceivably offering a more efficient carrier delivery from bulk to depletion region. The overall result of a combination of unfavorable weak light adsorption and favorable diffusion length is that most of the incident photons interacting with WO_3_ photoanodes are absorbed beyond the depletion region, resulting in an insufficient charge carrier separation despite the carrier's high mobility.

In the past WO_3_ photoanodes were usually prepared as thick films (typically a few micrometers in thickness) to overcome the weak visible light absorption.^3–13^ Other attempts to improve absorption included tuning the illuminated surface area by introducing surface microstructures,^6,14–17^ which might not be a practical and scalable way forward. In addition to tuning light absorption on photoanode, another strategy to improve the photocurrent is to overcome the kinetic limitation in O_2_ evolution by introducing modification on the photoanode surface with appropriate co-catalysts. Among a few examples of oxygen evolution catalysts deposited on WO_3_, Bi_2_S_3_, Sb_2_S_3_, CoO_*x*_, FeOOH, and IrO_2_ are the most promising ones, with only IrO_2_ offering good stability in acidic electrolyte.^18–24^ While exhibiting an improved oxygen evolution performance, these catalysts also reduce the photon flux reaching WO_3_ due to their non-transparent nature.^25,26^ Therefore, the potential improvements for oxygen evolution by surface-decorated co-catalyst is offset by reduced light absorption. In addition to modifying surfaces, adding catalysts to electrolytes was also explored. It was found that both phosphotungstate and phosphomolybdate improved the WO_3_ photoanode performance.^27^ However, despite some success in using the above mentioned strategies, it is still a challenge to develop highly efficient visible light absorbing photoanodes with very transparent oxygen evolution catalysts that can also offer thermodynamic stability in acidic electrolytes.^18^

In order to move this field further, it is important to critically evaluate the mechanistic aspects of the current electrode design strategies. In our work, we address a specific challenge of improving the PEC activity of unmodified WO_3_ electrode without creating a thick film electrode geometry, which causes photon adsorption beyond the carrier diffusion region. Introduction of oxygen vacancies to the surface of semiconductors can be one promising strategy to improve WO_3_ photocatalytic efficiency while keeping relatively thin thickness.^28^ Oxygen vacancies typically act as donor defects for n-type oxides, where they exert a significant influence on space charge region (SCR) and charge recombination.^11–13,29^ In addition, dual oxygen and tungsten vacancies have proven to significantly enhance the hole transfer efficiency at semiconductor electrolyte interface (SEI).^4^ Therefore, modulating the functions of vacancies at the surface of semiconductors using a finely controlled method can be a potentially promising way forward to design high performance thin film PEC electrodes. The novel aspects of this study are in developing sophisticated performance tuning of WO_3_ electrodes by simultaneous fine adjustment of both oxygen vacancies and thickness of the oxide layer, while maintaining a good crystallinity of the sample. Here we introduce a simple surface reduction method that can significantly enhance the performance of bare WO_3_ thin film photoanodes, which are only 500 nm thick. These photoanodes showed a performance similar to that of other literature described WO_3_ photoanodes with few micrometers in thickness. Our results demonstrate that a sophisticated control of the electrode surfaces reduction is necessary to further optimize water oxidation activity of WO_3_ photoanodes.

## 2. Results and discussion

**Fig. 1:**
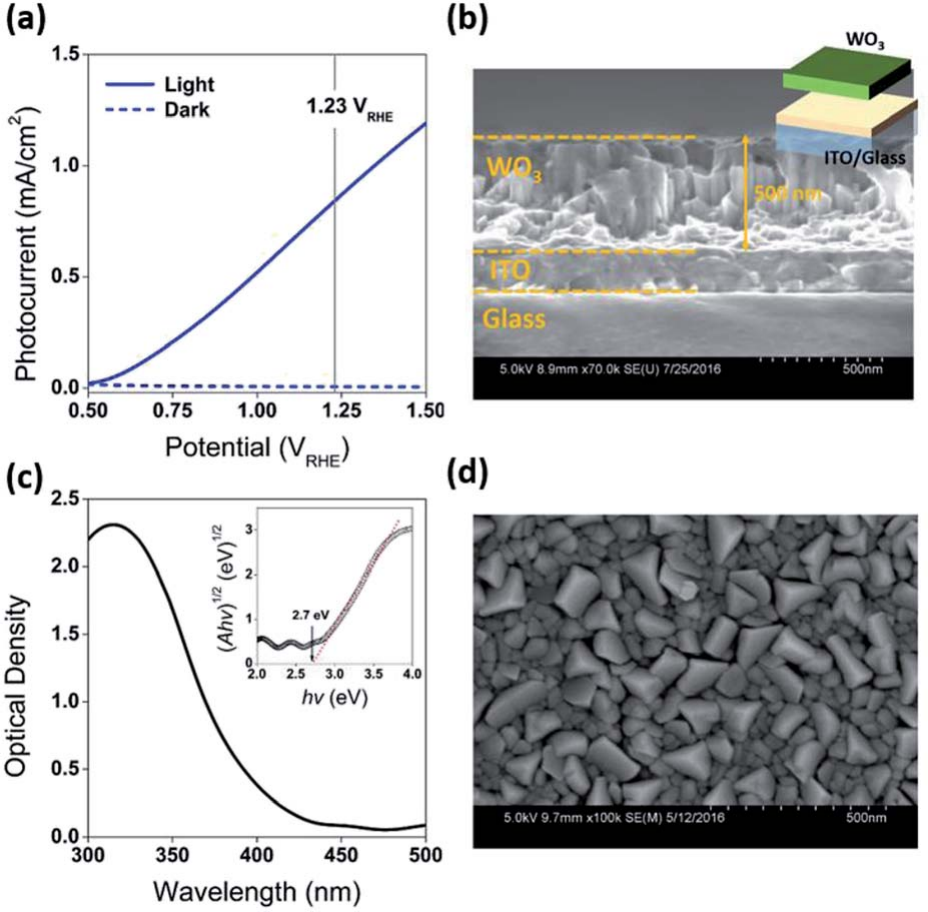
(a) Current density *vs.* potential under front-side AM 1.5 illumination for the as-prepared WO_3_ photoanodes (R0). The vertical line indicates the thermodynamic potential for oxygen evolution (1.23 *V*_RHE_). (b) Cross-sectional SEM image of WO_3_ thin film sample prepared by 40k laser pulses. (c) UV-vis spectrum of as-prepared WO_3_ photoanodes (R0) with Tauc plot inserted. (d) SEM image of as-prepared WO_3_ photoanodes (R0).

**Fig. 2:**
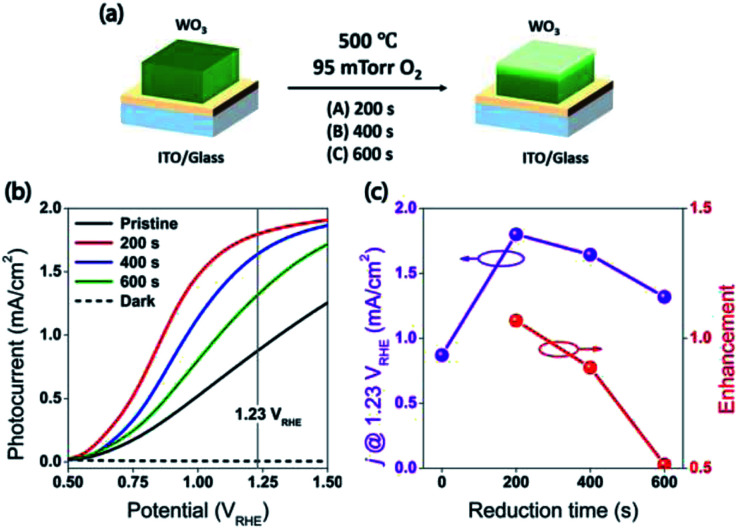
(a) The illustration of mild reduction treatment. (b) Current density *vs.* potential under front-side AM 1.5 illumination for the WO_3_ photoanodes, R0, R1, R2, and R3. The vertical line indicates the thermodynamic potential for oxygen evolution (1.23 *V*_RHE_). (c) The photocurrent of the WO_3_ photoanodes (R0, R1, R2, and R3) at 1.23 *V*_RHE_, and the relative enhancements for the reduced WO_3_ photoanodes (R1, R2, and R3). The characterization of the photoanodes were conducted in 0.5 M H_2_SO_4_ electrolyte.

Fabrication of the water splitting photoanodes was accomplished by depositing WO_3_ thin films on ITO glass substrates using pulsed laser deposition (PLD). The thicknesses of the WO_3_ thin films were controlled by the total number of laser pulses. The conditions of thin film deposition were optimized based on PEC measurements on as-deposited WO_3_ photoanodes (Fig. S2, ESI†). Following the PLD deposition, the film was annealed under decreased partial oxygen pressure (*p*O_2_) of 95 mTorr to achieve surface reduction (Fig. 2a). This approach was consistent with published strategy of adjusting WO_3−*x*_ composition by tuning (*p*O_2_) during annealing. Samples reduced for 0, 200, 400, and 600 seconds under 95 mTorr of *p*O_2_ were labelled as R0 (pristine), R1, R2, and R3 respectively. The as-deposited pristine WO_3_ film exhibited an intrinsic optical absorption up to 450 nm (Fig. 1c), which according to the Tauc plot indicates an indirect band edge at 2.7 eV. The X-ray diffraction (XRD) patterns collected from the thin films were indexed as monoclinic WO_3_ (PCPDF# 72-1465), and the strongest diffraction signal consistently arose from the (002) plane, indicating it as the preferred domain orientation (Fig. S4b, ESI†). The Raman spectra (Fig. S4c, ESI†) show the most intense bands at 805 cm^−1^ and 715 cm^−1^ were assigned to O–W–O stretching vibrations, while the bands at 274 cm^−1^ and 332 cm^−1^ were assigned to O–W–O bending vibrations.^30–33^ The scanning electron microscopy (SEM) image (Fig. 1d) showed high uniformity of the WO_3_ thin film morphology. The cross-sectional SEM image of WO_3_ thin film sample prepared by 40k laser pulses (Fig. 1b), which included ITO bottom electrode, indicated that its thickness was about 500 nm. After the reduction treatment, no significant differences in UV-vis, XRD and Raman of the reduced samples were observed (Fig. S4, ESI†). The lattice spacing calculated from the high-resolution TEM (HRTEM) images (Fig. 4c and f) was 3.90 Å, corresponding to the distance between the (002) planes of a monoclinic WO_3_ phase.

**Fig. 3:**
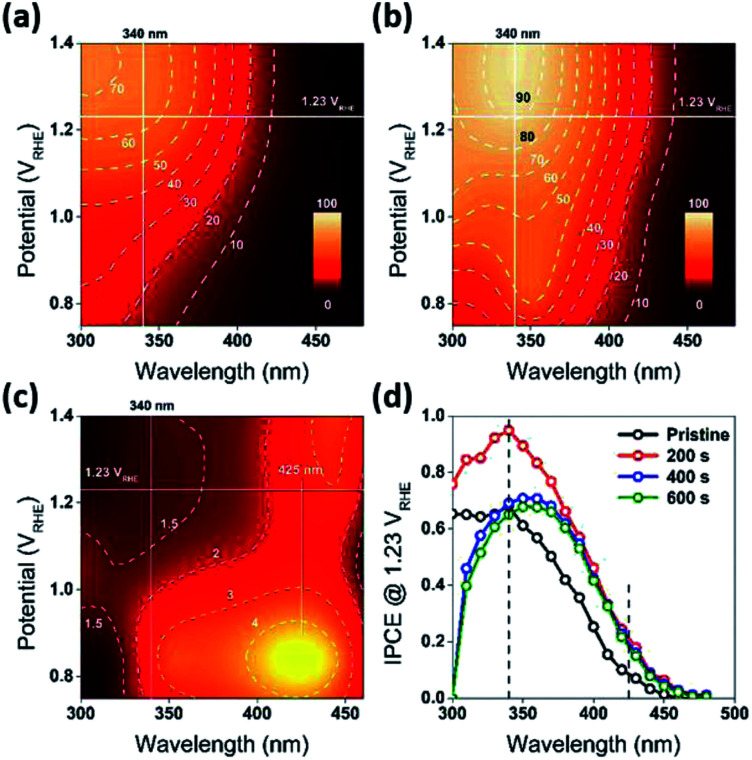
(a and b) Front-side (EE) IPCE as a function of excitation wavelength potential, for the R0 (Pristine) and R1 (reduced for 200 s) WO_3_ photoanodes. Both the panels share the same color scale. The dashed lines are contours for corresponding IPCE values in percentage. (c) The ratio of IPCE values for sample R1/R0. The dashed lines are contours for corresponding enhancement of IPCE values. The horizontal white lines indicate the thermodynamic potential for oxygen evolution (1.23 *V*_RHE_). (d) Front-side (EE) IPCE measurements for the WO_3_ photoanodes (R0, R1, R2, and R3), carried out at 1.23 *V*_RHE_. The characterization of the photoanodes were conducted in 0.5 M H_2_SO_4_ electrolyte.

The water splitting properties of the WO_3_ thin film photoanodes were studied by photoelectrochemical methods. According to the current density–potential (*J*–*E*) curves, which were measured in a 0.5 M H_2_SO_4_ solution (pH = 0.3) under front-side (electrolyte–electrode side, EE) AM 1.5G illumination (Fig. 2b and c), all the WO_3_ photoelectrodes produced anodic photocurrents that were consistent with the n-type doping. The onset potentials of all photoanodes were approximately 0.50 *V*_RHE_. The sample treatment in reduced atmosphere significantly improved photocurrents (samples R1, R2 and R3)., The sample R1, which was annealed for 200 s, exhibited the largest photocurrent (1.80 mA cm^−2^ at 1.23 *V*_RHE_), which was 2 times higher than that of the pristine sample. Increasing annealing time to 400 and 600 s, resulted in photocurrent decrease for samples R2 and R3. Additional insights into the observed trends were provided by the incident-photon-to-current efficiency (IPCE) measurements. Fig. 3 shows a dependence of the IPCE at 1.23 *V*_RHE_ on the excitation wavelength. A comparison between pristine (R0) and mildly reduced (R2) samples shows that R1 exhibited improved IPCE across the entire spectral range (300–480 nm). Fig. 3c shows the ratio of IPCE values for sample R1 : R0 as a function of the excitation wavelength and applied potential. It showed more than 4-fold enhancement in IPCE at 425 nm measured at lower applied potential. The maximum enhancement was up to 12 times at about 0.9 *V*_RHE_. In contrast to significant differences in the 300–340 nm spectral region between the sample reduced for the shortest period of time (R1) and the ones reduced for a longer time (R2 and R3), all the other samples showed a similar IPCE in the spectral range of 340–480 nm. Therefore, one can conclude that lower photocurrents for R2 and R3 samples as compared to that for R1 sample (Fig. 2b) can explain the sub-optimal performance of R2 and R3 electrodes (Fig. 3d) in the 300–340 nm spectral range. The results also suggest that there is an optimal duration of electrodes treatment in the reducing atmosphere that maximizes the IPCE in the visible light region.

**Fig. 4:**
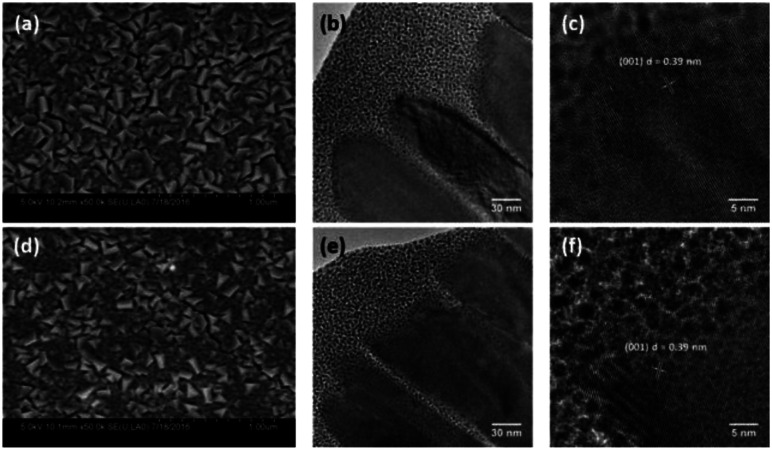
SEM and cross-sectional HRTEM (a–c), of pristine WO_3_ (R0) and (d–f), reduced WO_3_ (R3) photoanodes.

**Fig. 5:**
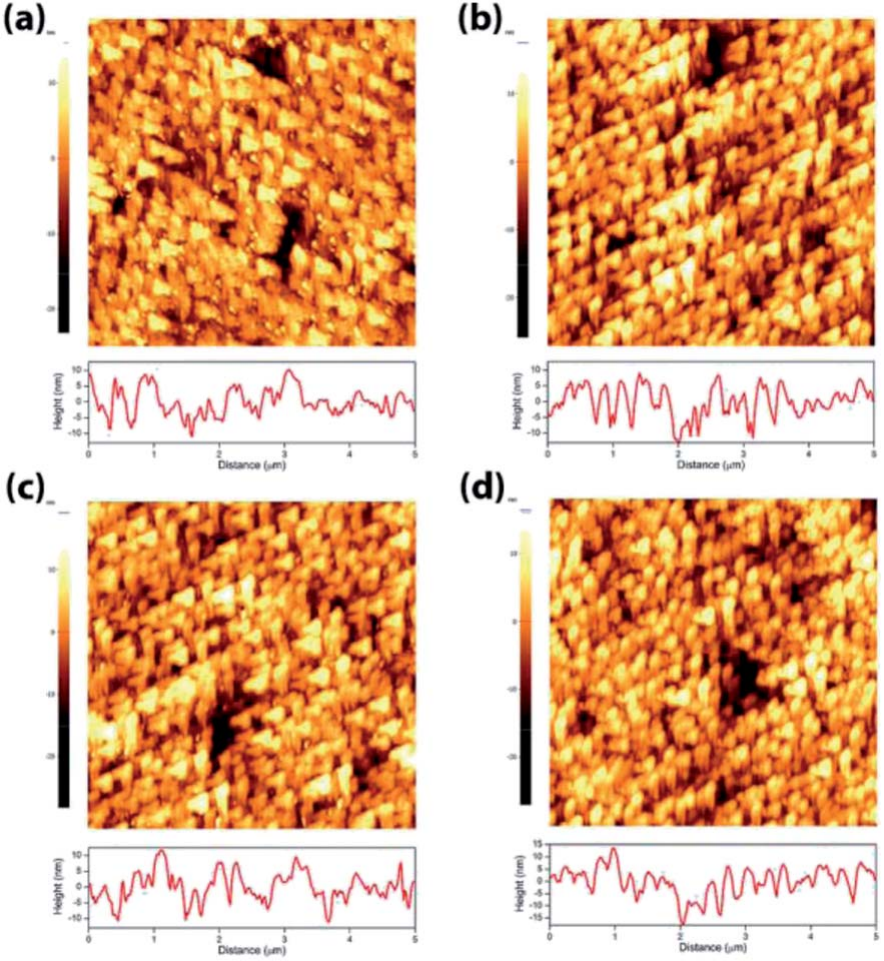
AFM for WO_3_ photoanodes (R0, R1, R2, and R3).

**Fig. 6:**
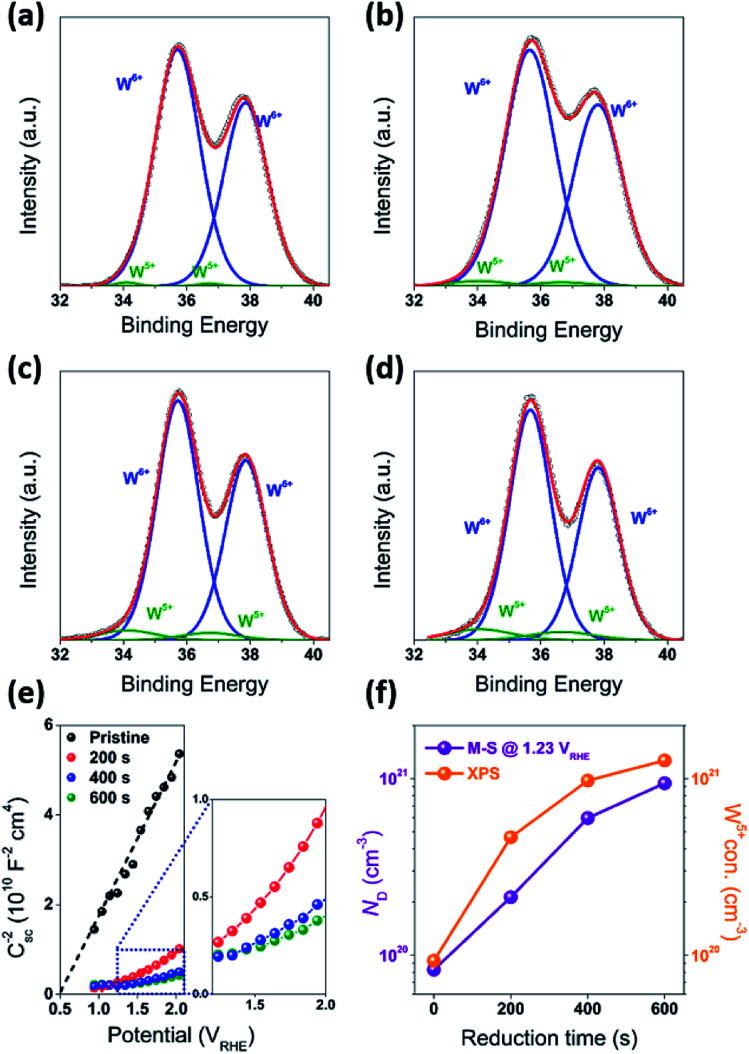
(a–d) W 4f XPS for WO_3_ photoanodes (R0, R1, R2, and R3). (e) Mott–Schottky plots, for pristine and WO_3_ photoanodes (R0, R1, R2, and R3). (f) *N*_D_ and W^5+^ concentration as a function of reduction time.

In order to understand the effect of electrodes' treatment on their structure, morphology and surface composition, a further characterization of the samples was performed by X-ray diffraction, X-ray Photoelectron Spectroscopy (XPS), Atomic Force Microscope (AFM) and Electrochemical Impedance Spectroscopy (EIS). No new X-ray diffraction peaks or significant peak shift in the existing peaks were observed for the reduced WO_3_ samples (Fig. S4a–c, ESI†), suggesting that the reduction treatment had a minimal effect on WO_3_ lattice structure. The surfaces of the pristine and reduced WO_3_ samples were further investigated by atomic force microscopy. The average surface roughness (*R*_a_) for R0, R1, R2 and R3 samples was 4.5, 4.2, 5.1, and 5.4 nm, respectively. Despite the fact that the surfaces of R0, R1, and R2 samples appeared to have very similar granular structures, these structures were not present in the R3 sample reduced for the longest time (Fig. 5a–d). These results suggest that the reduction treatment has some subtle effects on WO_3_ morphology, although defining the trends in morphological changes might require further investigation. The effect of sample reduction on the WO_3_ surface composition was further studied by the XPS in conjunction with EIS measurements. Fig. 6a–d shows high-resolution W 4f spectra of the WO_3_ photoanodes (R0, R1, R2, and R3). It can be clearly seen that the WO_3_ undergo significant evolution upon the reduction treatment. The W 4f doublet at 37.7 and 35.5 eV, can be assigned to W^6+^ species, while 36.5 and 34.3 eV doublet can be assigned to W^5+^ species. A comparison between W 4f peaks for samples reduced for different times (R1–R3) indicated an increased full width at half maximum (FWHM) of the peaks as compared to that for the pristine sample (R0). Such increase can be attributed to increased contribution of W^5+^ species to the overall spectra with increased reduction time (Fig. 6f). More specifically, the W^5+^ concentration was determined based on the ratio of W^6+^ and W^5+^ peak areas. The pristine sample (R0) had W^5+^ atomic concentration of 0.5%, (9.2 × 10^19^ cm^−3^, W^5+^ concentration in volume), while the most reduced sample (R3) had an increased W^5+^ atomic concentration of species of 6.8%, (1.26 × 10^21^ cm^−3^ W^5+^ concentration in volume). It is important to note that the presence of W^5+^ species is indicative of oxygen vacancies formation, which act as shallow donors for n-type WO_3_.^34^ In order to understand the effect of oxygen vacancies on PEC performance, the donor densities were determined by EIS, which measured the differential capacitance *C*_sc_ of the depletion region at various electrode potentials *E*. For a flat, uniformly doped semiconductor electrode, a linear relation between 1/*C*^2^_sc_ and *E* is defined by the Mott–Schottky equation:^24,35–37^1
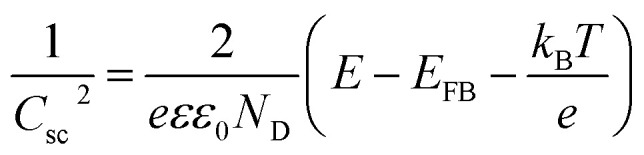


In our case, this equation was used to determine the donor density *N*_D_ based on such variables as elementary charge *e*, the dielectric constant *ε*, the vacuum permittivity *ε*_0_, the flat band potential *E*_FB_ and the thermal energy *k*_B_*T*. In our calculations the value of *ε* = 35 for the WO_3_ electrodes was based on the literature data.^15^ The *N*_D_ value for the pristine sample (R0) was 8.3 × 10^19^ cm^−3^ with *E*_FB_ = 0.5 *V*_RHE_, while *N*_D_ values increased monotonically with an increased exposure time to reducing atmosphere. More specifically, the *N*_D_ values for the reduced samples R1, R2, and R3 were 2.1 × 10^20^, 6.0 × 10^20^ and 9.4 × 10^20^ cm^−3^ respectively. It is useful to note that the donor densities determined by the EIS method were within one order of magnitude of W^5+^ surface densities determined by XPS, confirming a successful introduction of tunable concentration of oxygen vacancies into the WO_3_ surfaces by a very simple treatment.

To clarify the mechanism of the PEC enhancement by the mild reduction treatment, several factors have to be considered. Previously published modification of WO_3_ by various treatment methods introduced H_*x*_WO_3_ and/or WO_3−*x*_ (W^5+^ and oxygen vacancies) species into WO_3_ photoanodes, which resulted in the enhancement of PEC water oxidation.^4,28,38,39^ Although the studies mentioned above focused on photoanodes having different film thickness and surface pre-treatment conditions from those used in this work, it is still instructive to analyze these published results. More specifically the literature suggests that the WO_3_ photoanodes containing W^5+^ species are highly resistive to re-oxidation and peroxo-species induced dissolution.^28^ while exhibiting enhanced hole transfer efficiency.^4,38,39^ Our calculations based on Butler–Volmer model^40^ also showed that the mild reduction treatment on WO_3_ can also enhance the absolute magnitude of the electric field (*E*_0_) at the interface between semiconductor and electrolyte (Fig. S6a, ESI†), resulting in suppressing of electron collection efficiency (Fig. S6b, ESI†).^40^

**Fig. 7:**
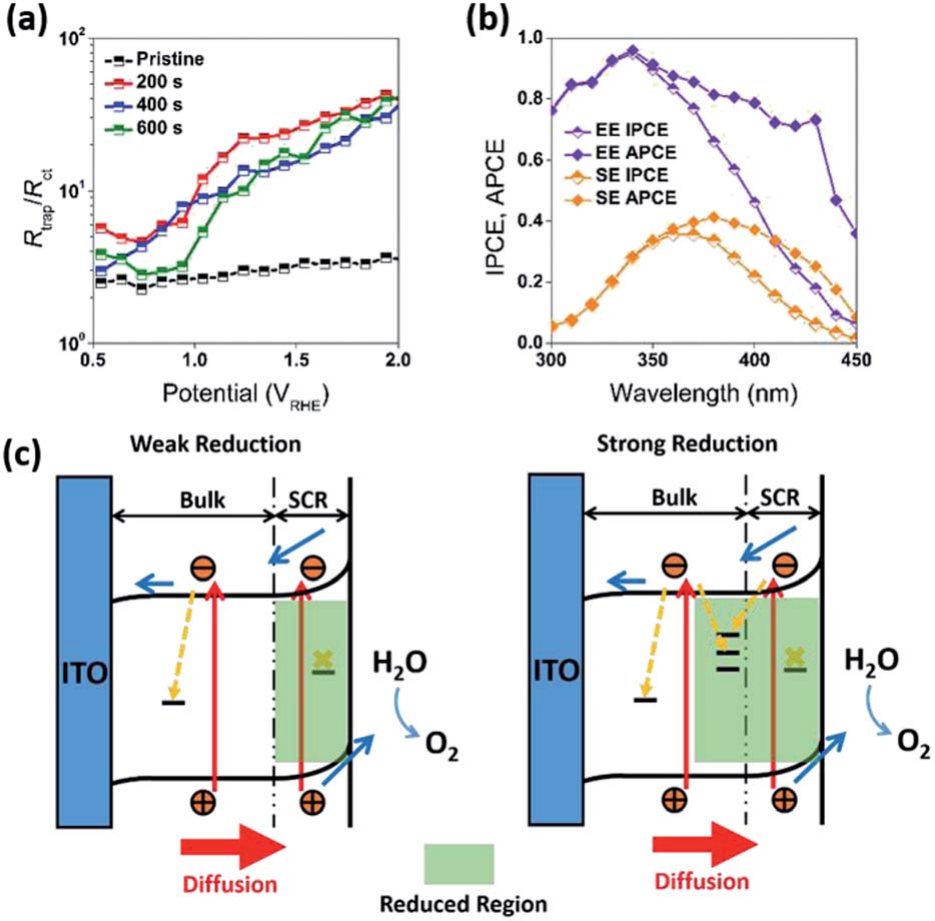
(a) *R*_trap_/*R*_ct_ for R0, R1, R2, and R3 photoanodes under front-side (EE) illumination. (b) Back-side (semiconductor–electrolyte side, SE) and front-side (EE) IPCE and APCE measurements for the optimized WO_3_ photoanodes (R1), carried out at 1.23 *V*_RHE_. The characterization of the photoanodes were conducted in 0.5 M H_2_SO_4_ electrolyte. (c) Schematic band bending diagram and charge transfer processes in WO_3_ photoanodes after weak and strong reduction treatments.

In addition to the above mentioned electronic effects, it is also important to consider such spatial factors as depletion width, effective minority carriers diffusion and the thickness of the higher dopant density region. These spatial factors can all be affected by the surface treatment. For example, reduction of the samples resulted in an increase in dopant concentration and decrease in a depletion width. At 1.23 *V*_RHE_, the calculated depletion width (*d*_sc_) of pristine WO_3_ (R0) electrode was about 7 nm, while the *d*_sc_ for the reduced WO_3_ (R1, R2, and R3) electrodes was about 2 nm (Fig. S6c, ESI†). In the classic Gartner model, photocurrent may only be produced when photogenerated charge carriers are created within both the depletion width and effective minority carrier diffusion length (*L*_p_). Given that the literature reported hole diffusion length for WO_3_ is *L*_p_ = 0.5 μm.^7,40,41^ while the measured sample thickness is also 0.5 μm, it is conceivable that all photogenerated holes were efficiently utilized as charged carriers that could diffuse into SCR. However, such efficient utilization of holes can't explain the difference in photocurrent and IPCE between R1, R2, and R3 electrodes, where the presence of the doped region did not improve the quantum efficiency or R2 and R3 samples across the entire spectrum. As evident from Fig. 3d, a thicker reduction region for R2 and R3 electrodes resulting from longer reduction times caused a precipitous decrease in IPCE within 300 nm to 340 nm wavelength window (Fig. 3d). To rationalize this dependence, it is important to emphasize that a mismatch between depletion and doped region widths is critical to explain such behavior. Considering that in our case the doped region is larger than the depletion region, the recombination sites for doped region of R2 and R3 electrodes most likely suppressed and quenched the photogenerated charge carriers excited by the shorter wavelength light in the 300–340 nm spectral region. However, for the mildly reduced WO_3_ photoanode (R1) it is conceivable that the reduction region has a better match to a depletion region. As result, the R1 electrode exhibited dramatic across 300–480 nm wavelength IPCE enhancement as compared to that for the pristine sample (Fig. 7c and d).

To get further understanding of the charge carriers' dynamic of at the SEI, photoelectrochemical impedance spectroscopy (PEIS) measurements were conducted at the PEC experimental conditions under illumination. These measurements allowed to model several aspects of an electrode behavior. As compared to the often used EIS equivalent model for dark condition (Fig. S5, ESI†), the equivalent circuit model utilized this our work (Fig. S7, ESI†) contains additional variables that reflect behaviors of the traps in photoanodes. These variables include a chemical capacitance (*C*_ss_) representing the chemical capacitance of the traps, a trapping resistance (*R*_trap_) representing the trapping/detrapping resistance of electrons that go from the conduction band to the surface traps, and a charge-transfer resistance (*R*_ct_) representing the resistance of hole transfer.^42,43^ The dependence of these variables (*C*_ss_, *R*_trap_, and *R*_ct_) on the applied potential measured for our samples is shown in the ESI (Fig. S7, ESI†). In order to get better insights from this dependence, it is informative to relate the hole collection efficiency to the ratio *R*_trap_/*R*_ct_,^44^ as following:2
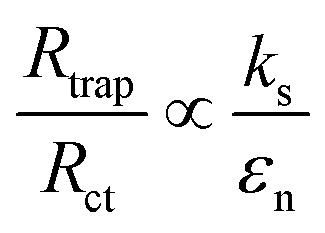
where *k*_s_ is the rate of charge transfer and *ε*_n_ is the rate of recombination. This ratio can also be used to reflect the kinetics of water oxidation at photoanode surface.^24,42,44,45^ As shown in Fig. 7a, the ratios of *R*_trap_/*R*_ct_ for pristine and reduced WO_3_ photoanodes generally increased as the applied potential increased, while the minimum values of this ratio were observed at the onset potential (0.5 *V*_RHE_) (Fig. 6a). As the applied potential increased, the ratios of *R*_trap_/*R*_ct_ for the reduced WO_3_ photoanodes (R1, R2, and R3) became more than one order of magnitude higher than that of the pristine WO_3_ photoanode (R0) (Fig. 7a). Among the three reduced samples (R1, R2, and R3), the R1 sample exhibited notably higher *R*_trap_/*R*_ct_ ratios than those for R2 and R3 samples. Such enhancement implies that a reduced WO_3_ surface tends to exhibit a higher rate of charge transfer. Moreover, the additional experiments on the R1 sample also indicated its high stability (Fig. S10a–c, ESI†). The observed trends in kinetics and stability are also consistent with previous studies demonstrating that a substoichiometric WO_3−*x*_ region at the WO_3_ electrode surface can dramatically enhance both photocurrent and photostability.^4,29,46^ This observation also suggests that by controlling the mild reduction duration, the charge transfer rate can be increased, which can positively contribute to the photocurrent and IPCE improvement.

## 3. Conclusion

In summary, WO_3_ thin film photoanodes were prepared by pulsed laser deposition under optimized conditions. The mild reduction treatment on WO_3_ photoanodes was controlled by post annealing in low oxygen partial pressure for different durations. The surface reduced of the samples was characterized by Mott–Schottky, XPS, PEC and IPCE measurements. The results work outlined the optimum conditions for the surface reduction treatment, as elevated level of defects beyond the space charge region resulted in loss of IPCE in UV region. The mildly reduced R01 sample exhibited largely improved PEC photocurrent density and IPCE across the entire 300–480 nm spectral window. This work provides a simple and well-defined roadmap for the preparation of high performance WO_3_ photoanodes by mild surface modulation. This approach might be also promising for other semiconductors with small dielectric constants, which can potentially include α-Fe_2_O_3_ and TiO_2_ based electrodes.

## 4. Experimental section

### 4.1 Thin film fabrication

WO_3_ thin films were grown by pulsed laser deposition (PLD/MBE-2300, PVD Products) on indium tin oxide (ITO) glass substrates (Thin Film Devices, 20 Ω sq^−1^). More specifically, a ceramic WO_3_ target was prepared by cold-pressing WO_3_ powder into a cylindrical pellet, which was finally sintered at 1100 °C for 12 hours. The WO_3_ target was ablated by KrF excimer laser pulses (*λ* = 248 nm) at a fixed repetition rate of 5 Hz, with a laser fluence of 1.5 J per cm^2^ per pulse. The substrate was placed 60 mm away from the target and heated to 500 °C by a non-contact radiative heating system under a controlled oxygen pressure (10–300 mTorr). The deposition rate for WO_3_ was about 0.15 Å per pulse. After the deposition, the substrate was cooled down at a constant rate of 10 °C min^−1^ to a room temperature. The surface of the samples was reduced by post annealing at 500 °C under 95 mTorr oxygen pressure for 200–600 seconds. Samples reduced for 0, 200, 400, and 600 seconds were labelled as R0, R1, R2, and R3 respectively.

### 4.2 Characterization

The X-ray diffraction patterns were recorded by an Ultima III diffractometer with parallel beam optics using Cu-Kα radiation (*λ* = 1.54184 Å). Optical characterization was performed in a Lambda 950 UV-vis-NIR spectrometer (PerkinElmer) equipped with a 150 mm integrating sphere. Scanning electron microscopy images were obtained using a HITACHI 4800 SEM at an accelerating voltage of 10 kV. The cross sections of the samples were prepared by focused ion beam *in situ* liftout using an FEI Helios dual beam SEM-FIB system. The X-ray photoelectron spectroscopy was performed in an ultra-high vacuum (UHV) chamber equipped with a hemispherical electron energy analyzer (Thermo Fisher Scientific, Alpha 110) and an Al/Mg twin anode X-ray source (Thermo Fisher Scientific, T352/NT).

### 4.3 Photoelectrochemical measurements

PEC measurements were performed in a custom-built, three-electrode cell with a quartz viewing window. The cell included a working photoanode, an Hg/Hg_2_SO_4_/saturated K_2_SO_4_ reference electrode (0.640 *V*_NHE_, WPI Inc.), and a platinum wire counter electrode. A 1.0 cm^2^ area of the working electrode was exposed to the electrolyte and illuminated from the front-side through the quartz window or the back-side through the ITO glass. The electrolyte was an aqueous solution of 0.5 M H_2_SO_4_ (pH ∼0.3). The linear sweep voltammetry (LSV) was performed with a sweep rate of 50 mV s^−1^ under dark or under simulated AM 1.5G illumination, using a Newport-Oriel 150 W Xenon arc lamp fitted with an AM 1.5G filter. The incident illumination power of 100 mW cm^−2^ was measured by a calibrated Si detector (Newport). The potentials referenced to the Hg/Hg_2_SO_4_ reference electrode were converted to potentials referenced to the reversible hydrogen electrode (RHE) using the Nernst equation,

3 *E*_RHE_ = *E*_Hg/Hg_2_SO_4__ + 0.0591*V* × pH + 0.682*V*

The monochromatic illumination for the IPCE measurements utilized a 300 W xenon arc lamp (Newport) coupled to a 1/8 m grating monochromator (Newport CS130), which was equipped with order sorting filters. The incident power was measured with an optical power meter (Newport 1918 C) and a UV-enhanced Si photodiode sensor. The IPCE is calculated based on the following equation,4
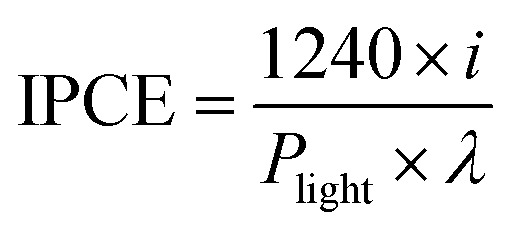
where *i* is the steady-state photocurrent density at a specific wavelength *λ* of the incident, and *P*_light_ is the light intensity for the specific wavelength (*λ*). The impedance of the electrochemical cell (*Z*) was measured under dark conditions at a series of electrode potentials, in the frequency (*f*) range 10^2^ to 10^5^ Hz.

## Conflicts of interest

There are no conflicts to declare.

## Supplementary Material

RA-009-C8RA08875F-s001
