# Supplementary material for: New aspects of improving the performance of WO3 thin films for photoelectrochemical water splitting by tuning the ultrathin depletion region
Source: RSC Adv. 2019 Jan 8;9(2):899–905. doi: 10.1039/c8ra08875f (PMC9059637; doi:10.1039/c8ra08875f)
Supplement: RA-009-C8RA08875F-s001 [file RA-009-C8RA08875F-s001.pdf]

Electronic Supplementary Material (ESI) for RSC Advances.
This journal is © The Royal Society of Chemistry 2018

Supporting information

New Aspects of Improving the Performance of WO_3 Thin Films for Photoelectrochemical Water Splitting by Tuning the Ultrathin Depletion Region

Jiajie Cen,[a] Qiyuan Wu,[a] Danhua Yan,[ab] Wenrui Zhang,[b] Yue Zhao,[a] Xiao Tong,[b] Mingzhao Liu,*[b] and Alexander Orlov*[a]

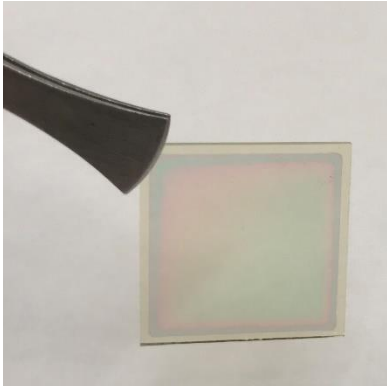

Figure S1. Photograph of the WO_3 thin film photoanode prepared *via* PLD.

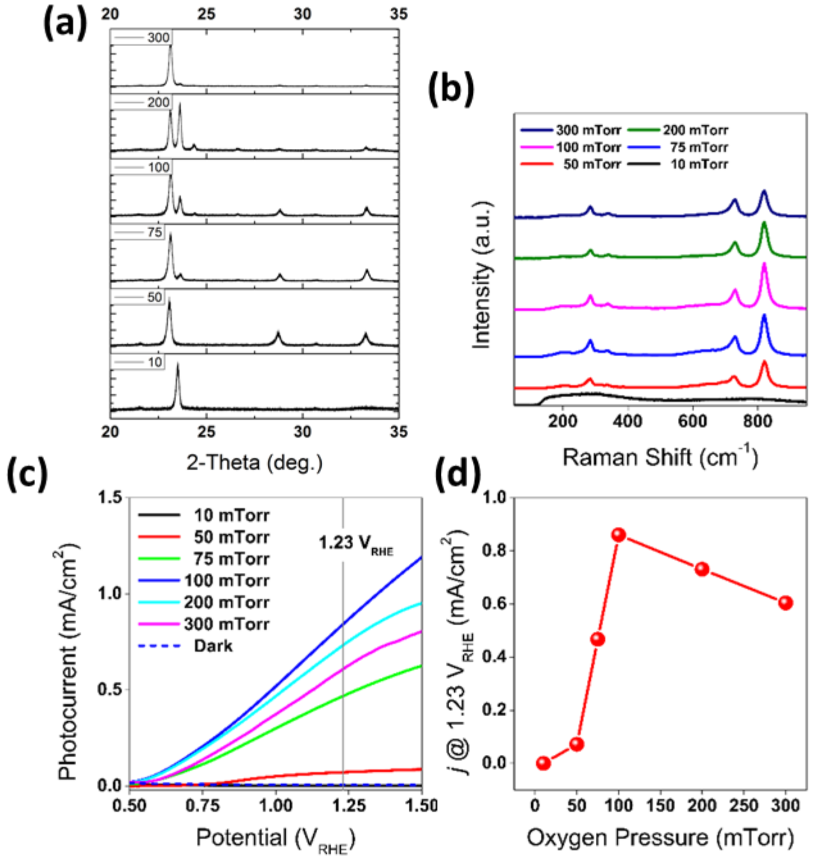

Figure S2. a, XRD, **b,** Raman, **c,** LSV, and **d,** photocurrents at 1.23 V_{RHE} for the WO_3 thin film samples prepared under different oxygen partial pressures.

The WO_3 thin films showed different preferential orientations when they were prepared under different partial pressures. When the oxygen pressure was higher than 10 mTorr, the crystallinity was optimized, which was confirmed by Raman. When the oxygen partial pressure was at 100 mTorr, the photocurrent became the highest.

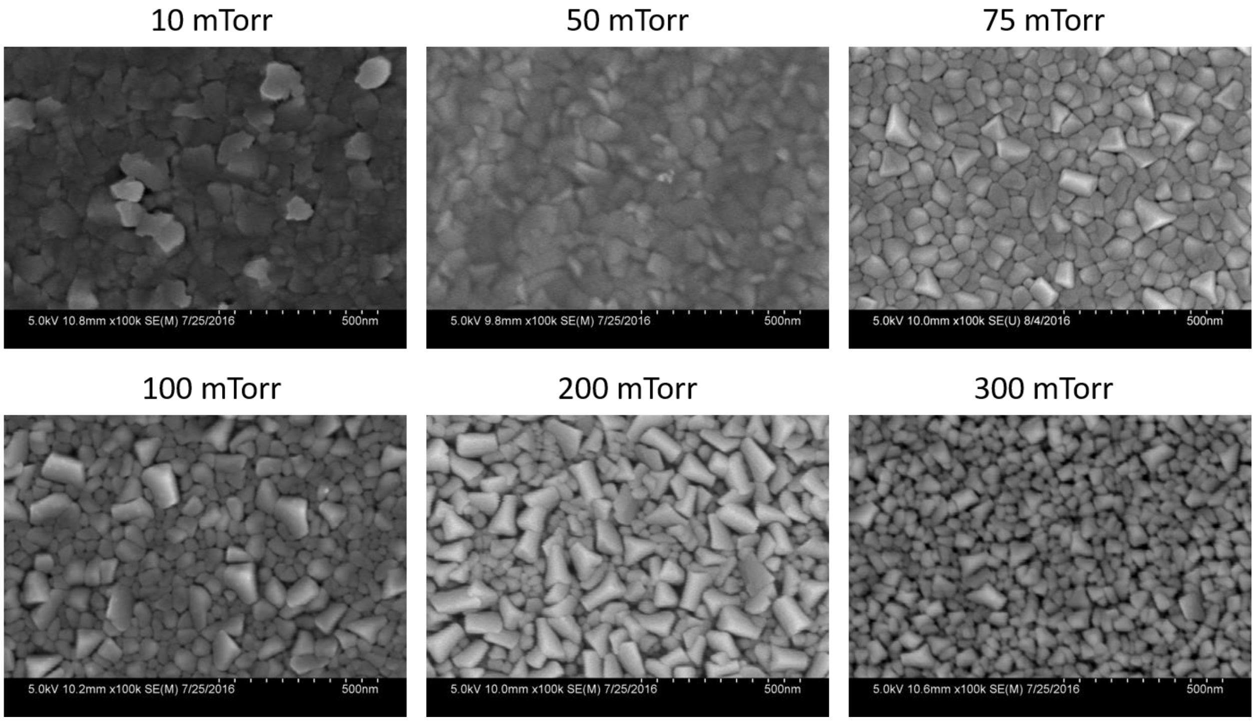

Figure S3. SEM images for the WO_3 thin film samples prepared under different oxygen partial pressures.

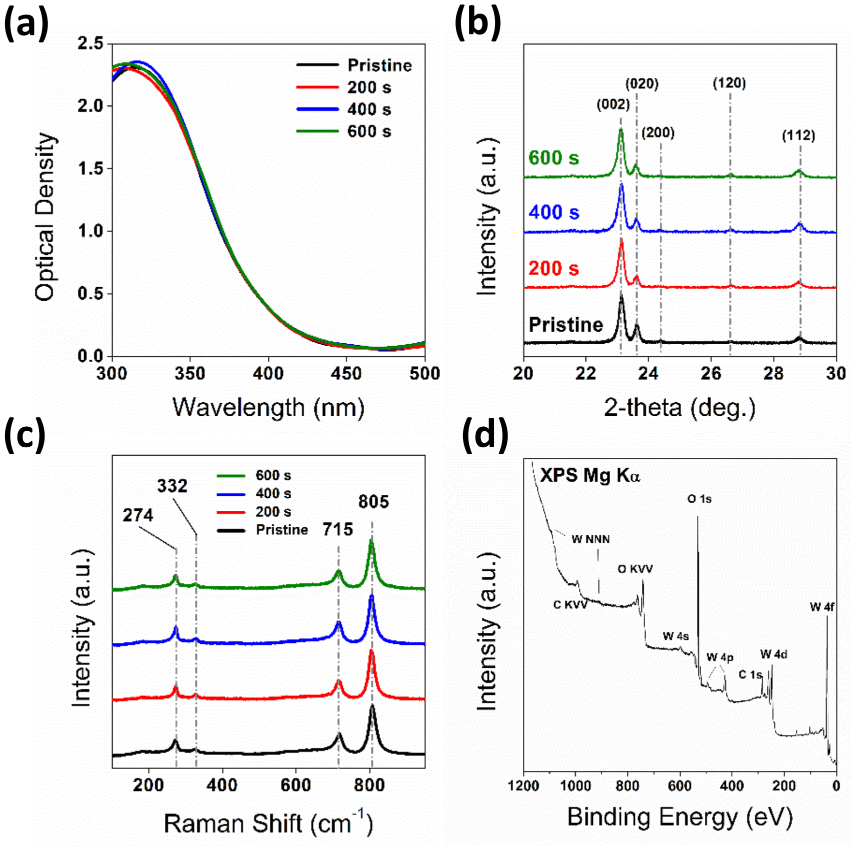

Figure S4. **a,** UV-vis spectrum of the pristine WO_3 photoanode. **b**, XRD patterns, **c,** Raman spectra of the pristine WO_3, and reduced WO_3 samples (R-0, R-1, R-2, and R-3). **d**, XPS survey for a WO_3 thin film sample.

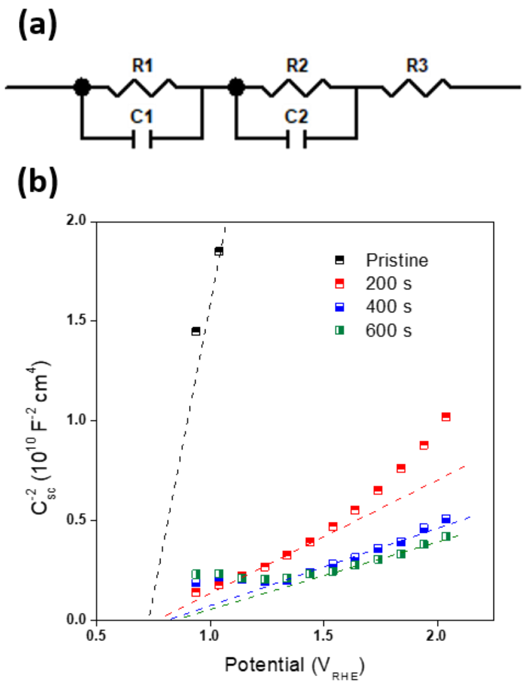

Figure S5. (a) The equivalent circuit used to model the electrochemical impedance of the cell. (b) Flat band indications by EIS results. R1, R2, and R3 are about 0.77, 0.79, and 0.81 VRHE respectively. These values are in the range of the published results. [1-4]

The impedance of the electrochemical cell (Z) was measured under dark conditions at a series of electrode potentials, in the frequency (*f*) range 10^2 to 10^5 Hz. To extract the space-charge region capacitance from the experimental impedance, the electrochemical cell was modeled by the equivalent circuit shown in Figure S6. The two current components flow together through the electrolyte solution and the electrical contact to the working electrode, which were represented by a series resistance *R*3. The current passing through the cell contributes in parallel to the faradic process that is represented by the charge transfer resistance R_2 and nonfaradic interface charging that is represented by *C*2 (C_{sc}), the capacitance per unit area of the aqueous interface. The capacitance *C* includes the space-charge region capacitance *C*2 (C_{sc}) and the double-layer capacitance *C*1 (C_{dl}) in series, $1/C = 1/C_{sc} + 1/C_{dl}$. The experimental data shown in Figure 5a is then fit using the EIS Spectrum Analyzer (ZVIEW2), software for analysis of impedance spectrum.

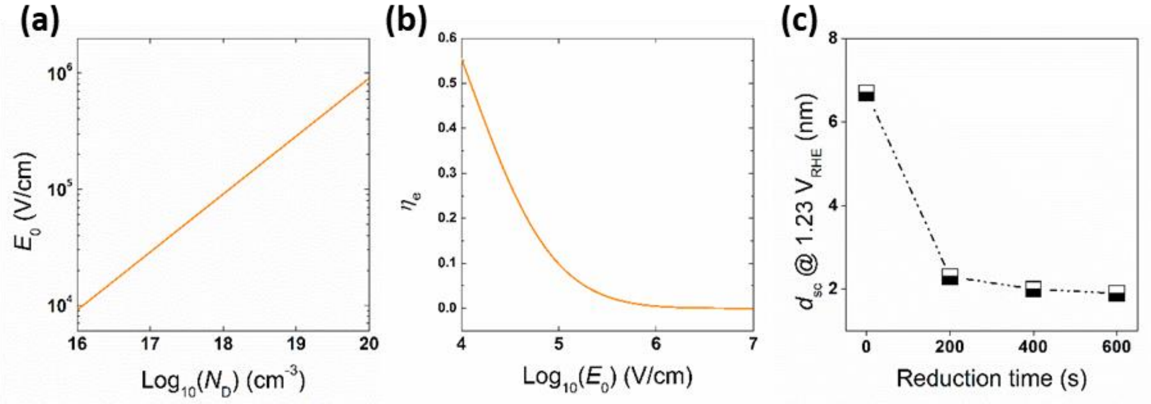

Figure S6. a, the absolute magnitude of the electronic field at the interface E_0 as a function of the donor density N_D. **b**, the electron collection efficiency η_e as a function of the absolute magnitude of the electronic field at the interface. **c**, thickness of the depletion region (d_{sc}) as a function of reduction time.

Given that the photogenerated majority carriers can diffuse in opposition to the electric field to the interface and therefore reduce the photocurrent. To describe this behavior, the electron collection efficiency can be determined by the following equation,

$$\eta_{\mathrm{e}} = \frac{1}{(1 + \frac{\mu_n E_0}{\upsilon_c})(1 + \frac{E_0 e}{\alpha kT})}$$

where μ_n is the election mobility, E_0 the absolute magnitude of the electronic field at the interface, υ_c the effective collection velocity, and e, α, kT as the previous definition. This expression represents the collection efficiency for photogenerated elections that diffuse to the interface rather than drift to the back-contact side. In the depletion approximation, the absolute magnitude of the electronic field at the interface E_0 is given by,

$$E_0 = \sqrt{\frac{2eV_{\mathrm{bi}}N_{\mathrm{D}}}{\varepsilon\varepsilon_0}}$$

where e, ε, ε_0, and N_D follow the previous definition, and V_{bi} the built-in voltage. Therefore, by increasing donor density N_D, E_0 can be increased with pertinence. Figure S6a showed that E_0 can be enhanced significantly with enhanced N_D for the WO_3 photoanodes after reduction treatment. Therefore, the electron collection efficiency can be reduced by increasing the donor density. By the mild reduction in our case (Fig. S7b), the electron collection efficiency can be suppressed effectively.

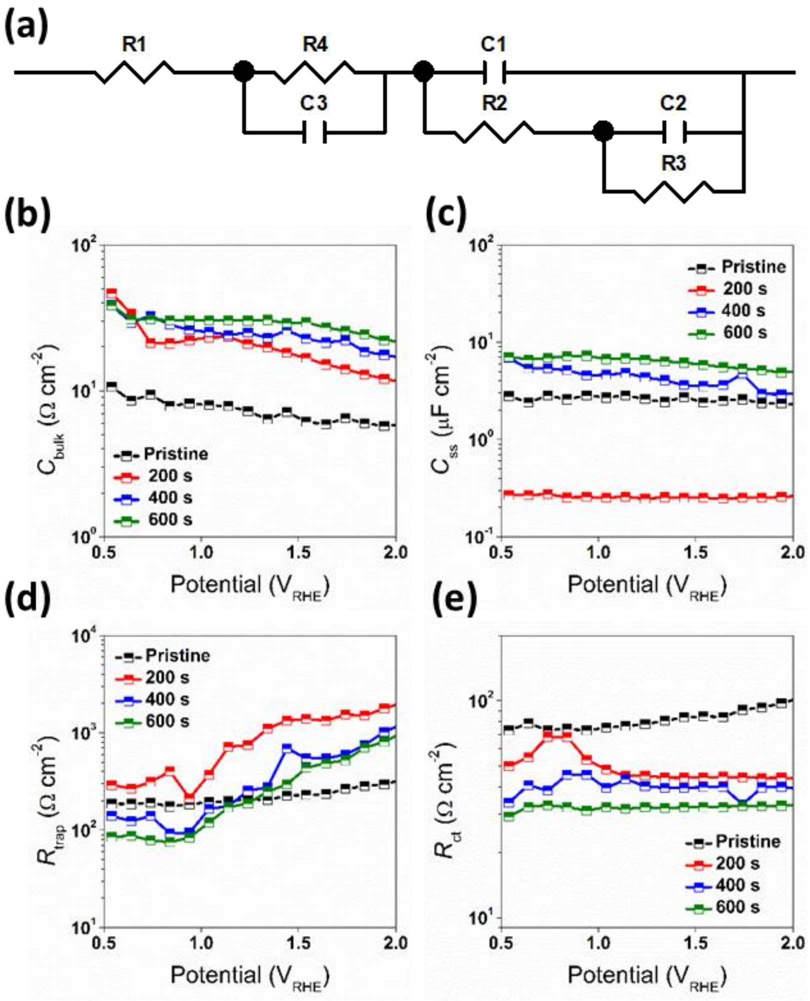

Figure S7. Equivalent circuits used to fit the experimental EIS data under illumination and the impedances and resistances parameters extracted from the fitting.

Compared to the EIS equivalent model for dark condition (Fig. S5, ESI), this equivalent circuit model (Fig. S7, ESI) additionally contains a chemical capacitance (C_{ss}) representing the chemical capacitance of the traps, a trapping resistance (R_{trap}) representing the trapping/detrapping resistance of electrons that go from the conduction band to the traps at the surface, and a charge-transfer resistance (R_{ct}) from the traps.

The current passing through the cell contributes in parallel to the faradic process that is represented by the serial resistance $R1$, trapping resistance $R2$ (R_{trap}), charge transfer resistance $R3$ (R_{ct}) and nonfaradic interface charging that is represented by $C1$ (C_{bulk} or C_{sc}), double layer capacitance $C3$ (C_{dl}), surface trap capacitance C2 (C_{ss}).

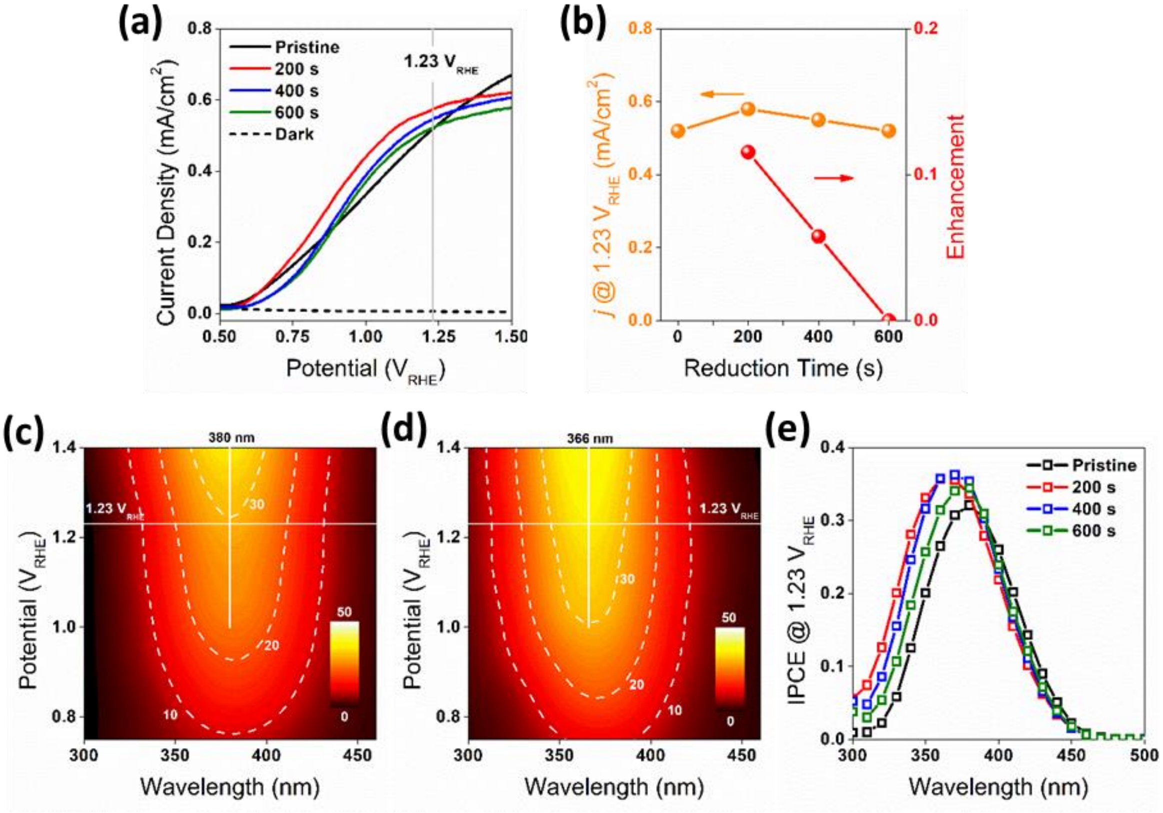

Figure S8. a, Current density vs. potential under back-side (SE) AM 1.5 illumination for the WO_3 photoanodes R-0, R-40, R-60, and R-80. The vertical line indicates the thermodynamic potential for oxygen evolution (1.23 V_{RHE}). **b,** The photocurrent of the WO_3 photoanodes (R-0, R-1, R-2, and R-3) at 1.23 V_{RHE}, and the relative enhancements for the reduced WO_3 photoanodes (R-1, R-2, and R-3). **c – d**, back-side (BE) IPCE as a function of excitation wavelength potential, for the R-0 (Pristine) and R-40 (reduced for 200s) WO_3 photoanodes. Both the panels share the same color scale. The dashed lines are contours for corresponding IPCE values in percentage. **e,** back-side (SE) IPCE measurements for the WO_3 photoanodes (R-0, R-40, R-60, and R-80), carried out at 1.23 V_{RHE}. The characterization of the photoanodes were conducted in 0.5 M H_2SO_4 electrolyte.

The *J* – *E* curves under illumination from the back of the WO_3 photoanodes R-0, R-1, R-2, and R-3 (back-side, SE) were plotted in Fig.5a. Different from the *J* – *E* curves under EE illumination, the photocurrents of each WO_3 photoanodes (R-0, R-1, R-2, and R-3) were much lower than that of the photoanodes under SE illumination, and they were all close to 0.5 ~ 0.6 mA/cm^2 at 1.23 V_{RHE}. For example, the photocurrent of R-1 at 1.23 V_{RHE} was only 0.57 mA/cm^2, slightly higher than that of R-0. The photocurrents of R-2 and R-3 at 1.23 V_{RHE} decreased as the reduction time increased. The subsequent IPCE measurements was plotted in Fig. 5e with back-side (EE) IPCE at 1.23 V_{RHE} versus excitation wavelength. The IPCE of the reduced WO_3 photoanodes (R-1, R-2, and R-3) presented significant enhancement in the range from 300 to 360 nm. However, in the range from 360 to 500 nm, the IPCE for all the four WO_3 photoanodes (R-0, R-1, R-2, and R-3) did not present obvious differences. Nonetheless, the SE and EE PEC performances and IPCE attributable to excitation from the wavelengths did not exhibit a simple correction with the reduction time on the WO_3 photoanodes, which, however, is consistent with the XPS and Mott-Schottky study analysis described below.

The back-side (SE) IPCE presented no enhancement in the longer wavelength range from 360 to 500 nm. This resulted from the recombination sites in the subsurfaces beyond the SCR for R-1, R-2, and R-3 suppressed and quench the photogenerated charge carriers excited by the longer-wavelength light 360 – 500 nm coming from the back of the electrodes. Given that enhancement of the SE IPCE in 300 – 360 nm was not significant, the photocurrents of R-1 – R-3 could not be promoted due to the very small portion of UV after AM 1.5 filter. In the case of R-3 under SE illumination, the enhancement of the IPCE in short wavelength could barely compensate the loss of that in wavelength longer than 360 nm, which overall presented as close photocurrent between R-0 and R-3.

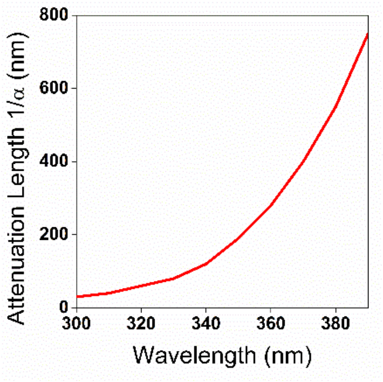

Figure S9. The attenuation length as a function of wavelength for WO_3, regenerated from references 1[5] and 2[6].

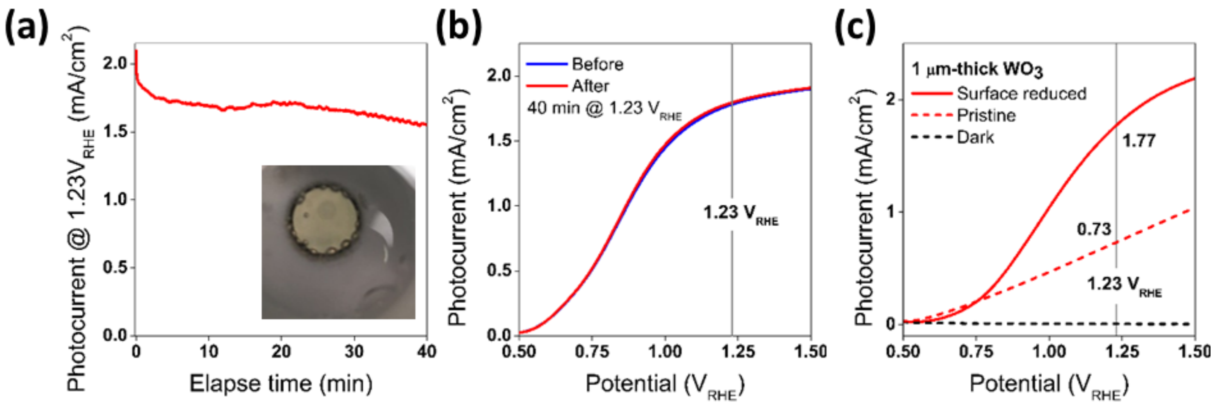

Figure S10. a, stability testing of the reduced WO_3 sample (R1). **b,** LSV for R1 before and after the stability testing. **c,** LSV for Reduction-200 s treatment on a WO_3 with a thickness of 1 μm.

Reference

1. Y. Liu, J. Li, W. Z. Li, Y. H. Yang, Y. M. Li and Q. Y. Chen, *Journal of Physical Chemistry C*, 2015, **119**, 14834-14842.
2. Y. Liu, J. Li, H. Tang, W. Z. Li, Y. H. Yang, Y. M. Li and Q. Y. Chen, *Electrochemistry Communications*, 2016, **68**, 81-85.
3. Y. Liu, Y. Yang, Q. Liu, Y. Li, J. Lin, W. Li and J. Li, *J Colloid Interface Sci*, 2018, **512**, 86-95.
4. Y. Liu, Y. H. Yang, Q. Liu, H. Z. He, W. H. Liu, D. D. Meng, Y. M. Li, W. Z. Li and J. Li, *Int J Hydrogen Energ*, 2018, **43**, 208-218.
5. R. A. Pala, A. J. Leenheer, M. Lichterman, H. A. Atwater and N. S. Lewis, *Energy & Environmental Science*, 2014, **7**, 3424-3430.
6. G. A. Niklasson, J. Klasson and E. Olsson, *Electrochim Acta*, 2001, **46**, 1967-1971.
